# Experimental demonstration of a concave grating for spin waves in the Rowland arrangement

**DOI:** 10.1038/s41598-021-93700-z

**Published:** 2021-07-09

**Authors:** Ádám Papp, Martina Kiechle, Simon Mendisch, Valentin Ahrens, Levent Sahin, Lukas Seitner, Wolfgang Porod, Gyorgy Csaba, Markus Becherer

**Affiliations:** 1 Department of Electrical and Computer Engineering, Technical University of Munich (TUM), Munich, Germany; 2 Faculty of Information Technology and Bionics, Pázmány Péter Catholic University, Budapest, Hungary; 3 Department of Electrical Engineering, University of Notre Dame, Notre Dame, IN 46556 USA

**Keywords:** Engineering, Nanoscience and technology

## Abstract

We experimentally demonstrate the operation of a Rowland-type concave grating for spin waves, with potential application as a microwave spectrometer. In this device geometry, spin waves are coherently excited on a diffraction grating and form an interference pattern that focuses spin waves to a point corresponding to their frequency. The diffraction grating was created by focused-ion-beam irradiation, which was found to locally eliminate the ferrimagnetic properties of YIG, without removing the material. We found that in our experiments spin waves were created by an indirect excitation mechanism, by exploiting nonlinear resonance between the grating and the coplanar waveguide. Although our demonstration does not include separation of multiple frequency components, since this is not possible if the nonlinear excitation mechanism is used, we believe that using linear excitation the same device geometry could be used as a spectrometer. Our work paves the way for complex spin-wave optic devices—chips that replicate the functionality of integrated optical devices on a chip-scale.

## Introduction

Information processing in today’s computers is done almost exclusively by charges (electric currents). Photons, albeit they are ideal for information transmission, never became a mainstream technology for computing. Despite their numerous advantages, photonic devices have practical limitations: they are challenging to integrate on-chip and optical wavelengths (about a micrometer) are huge compared to nanoscale devices, limiting the scalability of any photonic interference-based device.

Spin waves are wave-like excitations in magnetic materials that travel via coupling between precessing magnetic moments. In our current investigation we consider spin waves in ferro-, and ferrimagnets, but we note that spin waves are shown to exist in antiferromagnets and paramagnets as well^1^. Their wavelength can be adjusted in a wide range (from several micrometers down to potentially nanometer scale), and they have an electronics-friendly frequency range (1–100 GHz)^2^. This makes spin waves attractive for on-chip applications, especially in wave-based microwave signal processing. They also interact with each other (scatter), and the resulting nonlinearity may enable general-purpose computation. Unlike electromagnetic waves that can propagate in vacuum, spin waves exist only in (magnetic) medium and one needs high-quality materials to achieve ideal conditions for propagation and also carefully designed waveguide structures to launch (and pick up) the waves. It has only recently become possible to demonstrate short-wavelength, long-distance propagation^3,4^, spin-wave equivalents of optical laws^5^ or larger-scale refractive devices using local heating^6^. Spin-wave variants of complex optical devices have been shown recently by experimental demonstrations^7,8^—including an anisotropic spectrometer design^9^.

A key element of such a spin-wave optics device is a source which launches coherent spin waves. In the simplest case, the source of spin waves can be a simple coplanar waveguide that is placed atop the magnetic film. For many device constructions, such a simple construction is insufficient. Microwave waveguides alone are fairly inefficient at short spin-wave wavelengths^10^ and they are limited to generation of plane wavefronts or curved wavefronts with relatively small curvature. For generation of short-wavelength spin waves or non-planar wavefronts, lithographically patterning the edge of the magnetic film is desirable. A periodically patterned edge can serve both as a wave source and a diffracting element.

For sake of completeness we note that there is a number of emerging physical phenomena that one, in principle may use as sources of spin waves, such as spin-transfer and spin-orbit torques^11,12^ or optical methods. Still, waveguide-based (Oersted-field-based) generation of spin waves is perhaps the most straightforward method to use in microwave devices.

The focus of the present paper is the experimental study of such a patterned edge as a spin-wave launcher. We use Focused Ion Beam (FIB) irradiation to write a high-resolution concave grating pattern in an yttrium iron garnet (YIG) thin film. The grating is used in the Rowland spectrometer arrangement, which is frequently used in optical and X-ray spectroscopy^13^. This arrangement does not require a separate lens component, and thus it is ideal for waves with limited propagation length. We experimentally demonstrate that the edge of a FIB-irradiated pattern in YIG generates a coherent spin-wave wavefront. Using time-resolved Magneto Optical Kerr Effect (trMOKE) imaging we found that the diffraction patterns closely match those expected from theory and micromagnetic simulations. An unexpected discovery was that in our experiments spin waves were not primarily excited directly by the field of the waveguide. Instead, spin waves were generated indirectly by the dipole fields of high-amplitude, nonlinear, and long-wavelength standing waves that developed behind the grating in the unirradiated area. This process has a significantly higher efficiency of spin-wave generation at a distance from the waveguide. However, this quasi-homogeneous oscillation behind the grating only forms at a sufficiently high amplitude and only if a single frequency component is applied.

## Spectral decomposition with a concave diffraction grating

**Figure 1:**
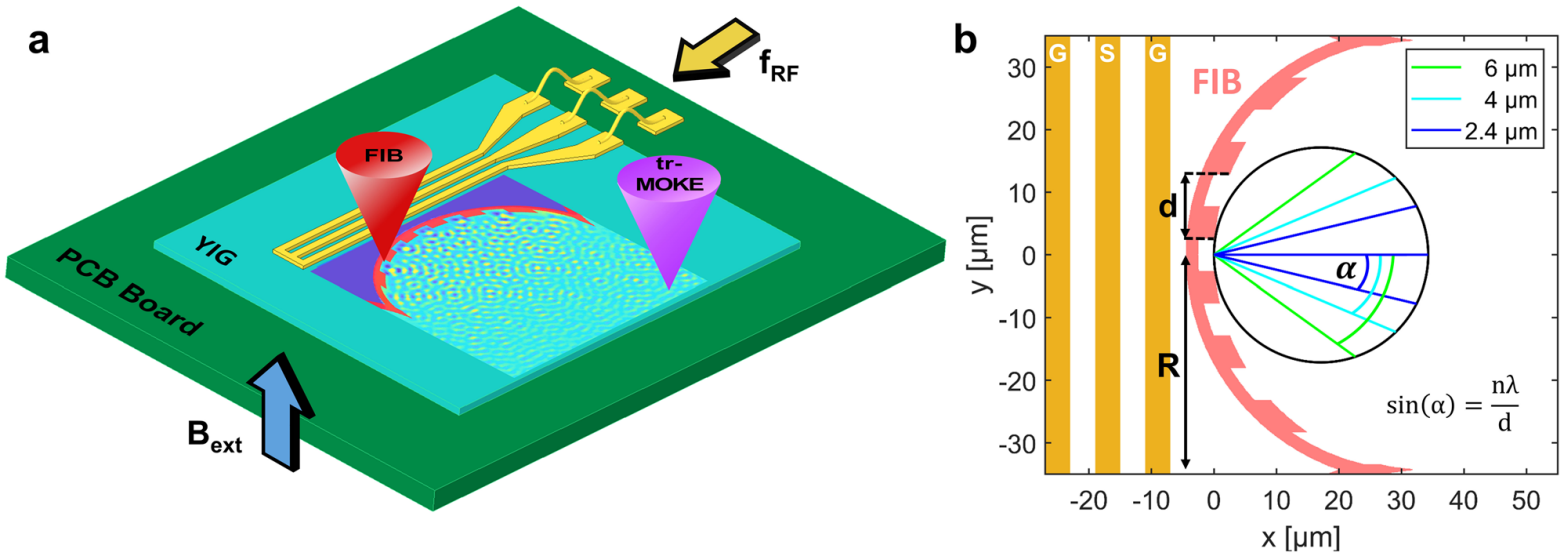
(**a**) Sketch of the experimental setup, indicating the coplanar waveguide, the FIB irradiated grating, the trMOKE probing laser, and the spin-wave interference pattern. (**b**) Geometry of the curved diffraction grating in the Rowland arrangement. The angle under which the first-order diffraction peak is seen from center of the grating is denoted by $\alpha$.

The main component of the proposed device is a concave diffraction grating that acts both as a wave source and as a diffractor. The fabrication of such a grating requires sub-wavelength patterning resolution as the pitch of the grating has to be comparable to the spin-wave wavelength. We use the so-called Rowland arrangement, a detailed description is given in^14^. The device generates a spectral decomposition of a time-domain signal by converting temporal frequency components to spatially separated spin-wave intensity peaks. The layout of the fabricated device is shown in Fig. 1. The microwave signal is converted to spin waves by a waveguide antenna. Each frequency component of the signal generates spin waves with corresponding wavelengths. Along the edge of the grating (FIB-irradiated region) the time-varying magnetic field is almost homogeneous, but due to the abrupt parameter change in YIG, every point along the edge acts as a wave source (also described in^15^). The curved grating not only diffracts different wavelengths to different directions, but also focuses the wavefronts. The drawing of Fig. 1b shows the geometry to determine the diffraction pattern. With a concave grating of radius *R* and ridge pitch *d*, the diffraction peaks (i.e. wavelength-dependent focal points) will form on a circle with radius *R*/2 drawn tangentially to the grating (Rowland circle). The *n*th-order diffraction angle can be calculated as $\alpha = \arcsin {\left( \frac{n\lambda }{d}\right) }$, where $\lambda$ is the wavelength of the spin wave.

In traditional optical or X-ray Rowland spectrometers the wave source is placed opposing the curved grating, which reflects the waves, acting as a secondary source. Such arrangement would be rather impractical for spin waves: the relatively long path between the source and the diffraction grating will cause much higher attenuation of spin waves. Thus, it is desirable that the grating and the source are in the same structure, i.e. the coherent spin-wave source itself is shaped as a curved diffraction grating. In this geometry, diffraction is caused by the phase difference between waves that originate from the bottom and the top of the ridges. This phase difference will also depend on the ratio of the ridge depth and the wavelength, which does not influence the diffraction angle, but it changes the relative amplitude between diffraction orders. In the designed structure the ridge depth also introduces an amplitude difference between waves that are generated on the top and the bottom of the ridge. The device is thus a combination of an amplitude grating and a phase grating.

**Figure 2:**
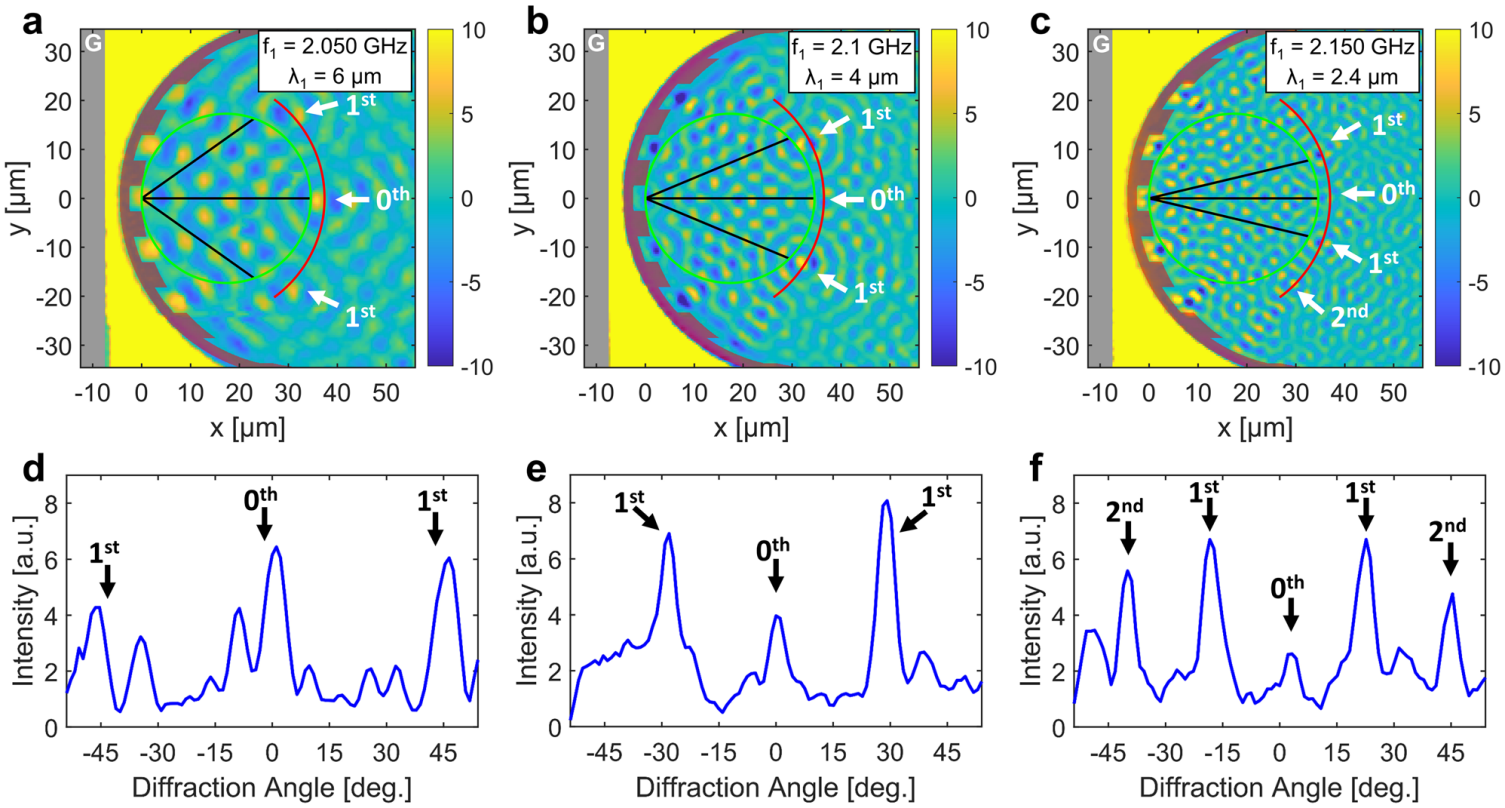
Spin-wave interference patterns recorded by trMOKE at three different excitation frequencies at constant bias field. In (**a**–**c**) the FIB irradiated region (grating) is indicated by a semi-transparent red overlay (radius R=30 µm, pitch d=12 µm), the gray stripe represents the ground line of the CPW. The green circles are the theoretical Rowland circles with a radius R/2, while the radius of red arcs are fitted to the data. Black lines indicate expected diffraction angles, and white arrows point to focal points in the measured data. The microwave power is kept constant at 5 dBm. (**d**–**f**) Spin-wave intensities extracted along the red arcs in (**a**–**c**).

We fabricated the designed device and recorded spin-wave interference patterns with trMOKE. The trMOKE images at three different excitation frequencies are shown in Fig. 2. Diffraction peaks are clearly observed close to the expected diffraction angles, as indicated by black lines. In case of the smallest wavelength the second-order peaks can also be observed ($\frac{2\lambda }{d} < 1$). We found that the peaks are not perfectly focused along the Rowland circle, but an arc can be fitted to the peaks that works well for all three wavelengths. We attribute this to the fact that our grating is much wider than conventional concave gratings ($180^\circ$ instead of a few degrees), and thus the approximations used in the derivation of the Rowland circle do not hold perfectly. This is confirmed by micromagnetic simulations, which also show that focal points are located on an arc with a slightly smaller curvature (see Fig. 4c). Another possible cause of deviations is the slight anisotropy of spin waves introduced by a tilt in the external bias field, which can not be fully eliminated in our current experimental setup.

We also note that side lobes (smaller peaks next to the labeled peaks) in Fig. 2 are due to the Airy pattern formation around the focal point (seen as circular pattern in the 2D scan). Since the size of the presented device is only an order of magnitude larger than the wavelength, these side lobes are filling a large portion of the output space, but with scaling up the device size compared to the wavelength this portion would decrease. Asymmetry of these peaks (e.g. in Fig. 2d) is probably due to fabrication defects.

## Generation mechanism of spin waves by nonlinear resonance

In most spin-wave devices, the magnetic field of the waveguide is directly responsible for launching the spin waves, as described in^14^. The relatively delocalized magnetic field of the waveguide and the localized demagnetizing field of the film edge jointly create a high, periodically changing torque and launch the spin waves^15^.

We found that in our device an indirect, nonlinear mechanism is dominant for spin-wave excitation. In the design presented in^14^ all material in the YIG film is assumed to be removed behind the grating. However, our FIB method is performed as the final fabrication step, after the CPW is already in place. We did not irradiate the total area between the grating and the CPW, only a narrow region, which is wide enough to block spin waves at the designed wavelength to significantly couple through via dipole fields. However, at sufficiently large excitation amplitudes the static (OOP) component of the magnetization decreases, which in turn decreases the demagnetization field of the YIG film^16^. This results in a higher effective field, and with that the spin-wave wavelength increases. The wavelength at sufficiently large amplitudes becomes much larger than the distance between the CPW and the grating and the spin waves that are generated under the CPW reflect back from the back side of the grating, creating a standing-wave pattern. Since the wavelength is much larger than the size of the region between the CPW and the grating, this resembles a homogeneous resonance in that region. This large area has a considerable oscillating dipole field that acts on the edge of the grating, launching secondary spin waves that form the Rowland interference pattern. We note that this mechanism does not change the frequency of the generated waves, only their wavelength in the strongly excited area behind the grating. One could refer to this effect as an amplitude-dependent ’refractive index’ in this region. Waves that are indirectly excited on the edge of the concave grating have the same frequency and wavelength as if they were generated by direct linear coupling from the waveguide. The trMOKE images of Fig. 2a–c already show the high-amplitude region on the left of the grating: it is observable that in this region the colormap is saturated and without apparent pattern, indicating large-amplitude, uniform precession. The applied microwave power was set to 5 dBm at the RF source, however the actual spin-wave amplitude depends on the frequency due to mismatches in the RF signal path and physical shape of the magnetic field of the CPW.

The dipole field of the quasi-homogeneous resonant excitation reaches significantly farther than that of the short-wavelength spin waves, thus it can excite coherent spin waves on the ridges of the grating. This field is in fact much stronger at this distance than the magnetic field of the CPW, becoming the dominant effect for spin-wave generation on the grating edge.

**Figure 3:**
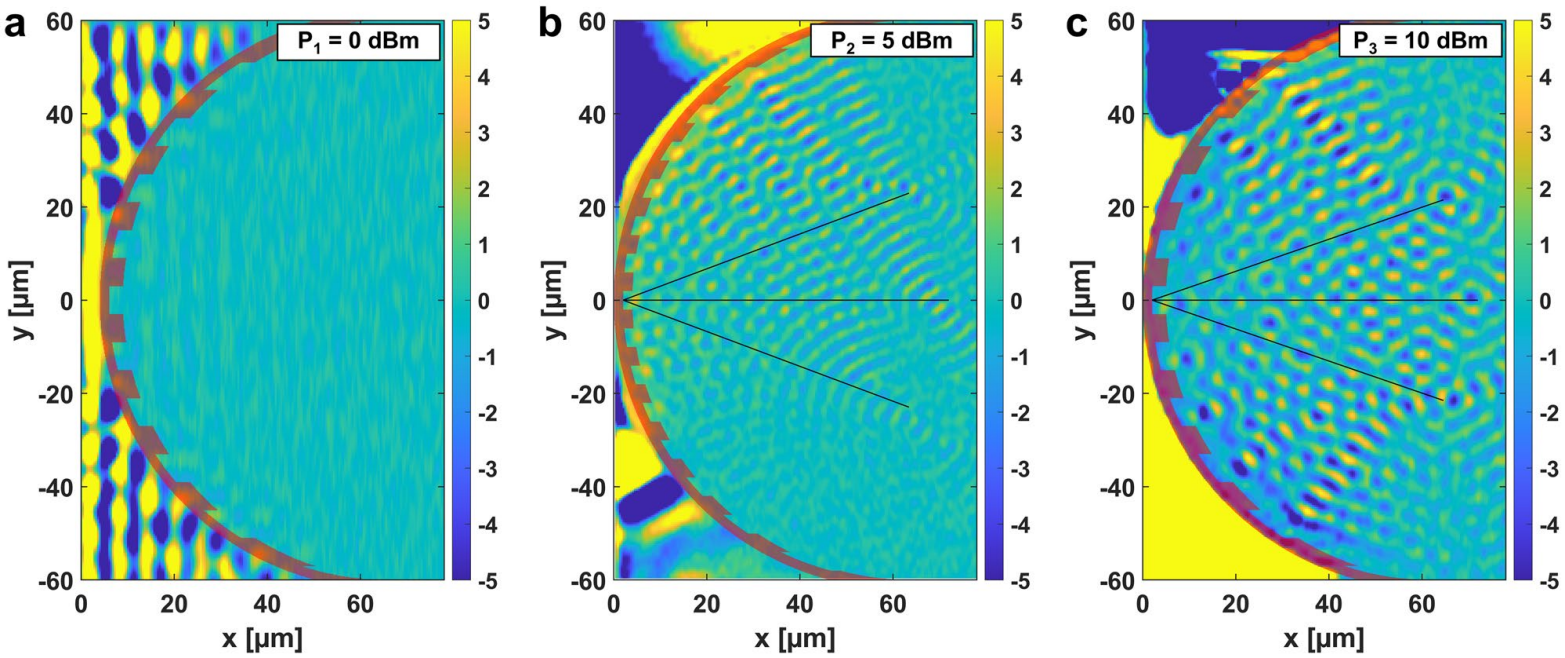
TrMOKE images of miscellaneous gratings expressing the difference in performance with respect to small (**a**), higher (**b**) and high (**c**) microwave current applied at the input waveguide. The right edge of the CPW ground line is 3 µm away from grating posterior in each case. The semi-transparent red overlay indicates the FIB-irradiated region, black lines show expected diffraction angles. The grating operation visibly enhances with the level of uniformity in the nonlinear spin wave excitation behind the FIB area.

Figure 3 shows the spin-wave-generation process in more detail. At sufficiently small excitation power ($P_{\mathrm {rf}} = 0$ dBm), linear spin waves are excited under the CPW. These small-amplitude, short-wavelength spin waves cannot significantly couple through the FIB-irradiated region. However, the Oersted field of the CPW is not sufficient to create spin waves at the grating edge, that could be detected by our trMOKE apparatus. In Fig. 3b ($P_{\mathrm {rf}} = 5$ dBm) nonlinear behavior is observable behind the grating, but the nonlinear wavelength is not yet long enough to create uniform precession. One can observe a partial interference pattern on the right possibly due to larger uniform standing waves on the top. At $P_{\mathrm {rf}} = 10$ dBm excitation power (Fig. 3c) the resonance is almost uniform (apart from the top region being out-of phase), and the interference pattern is complete. Here we note that although in Fig. 3c we used higher excitation power (10 dBm) than in Fig. 2 (5 dBm), we do not see fully homogeneous resonance behind the grating in the former case. This is mainly because in Fig. 3 we used a grating that is twice the size of the grating in Fig. 2, thus the distance and the area behind the grating is larger, requiring higher amplitude to fill. Also, the proportion power that reaches the antenna varies greatly from sample to sample, as fabrication variations and experimental conditions change transmission characteristics of the microwave path line. Thus, direct comparison of amplitude levels is not possible between different experiments.

**Figure 4:**
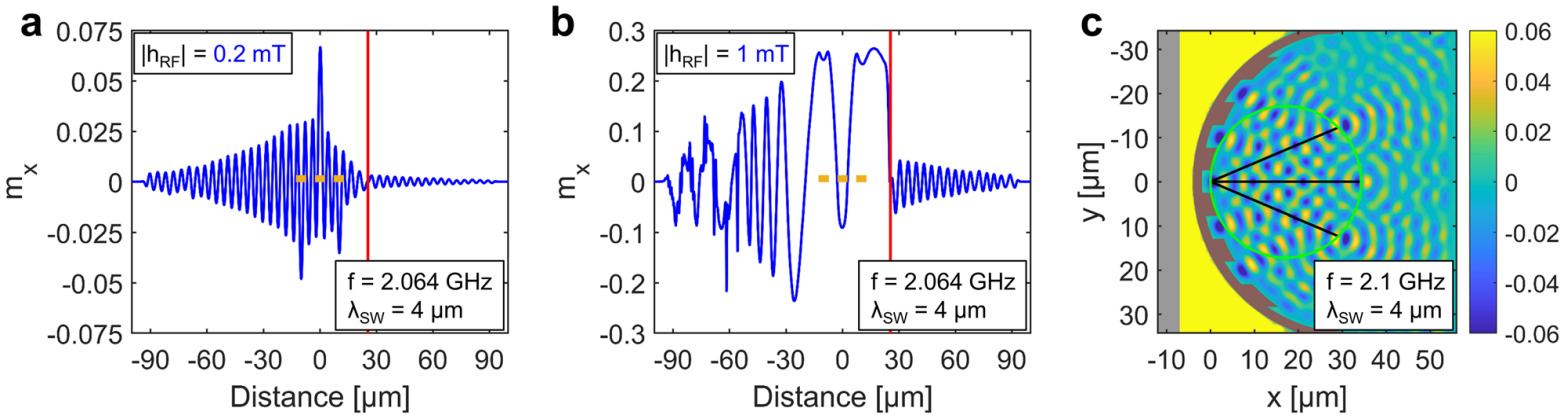
Micromagnetic simulations of nonlinear indirect excitation of spin waves. Yellow rectangles indicate the CPW position and width. The red stripe represents the FIB irradiated region, modelled by zero $\mathrm {M_s}$. (**a**) is a 1D example of linear excitation, while (**b** is a strongly nonlinear case. (**c**) represents a 2D simulation of the experimental scenario in Fig. 2b.

To gain more insight into the excitation process, we performed both one-dimensional and two-dimensional micromagnetic simulations using mumax3^17^. These simulations confirmed the behavior that we observed in the experiments (Fig. 4). At small excitation fields, spin waves beyond the grating are very small in amplitude (Fig. 4a). At a higher (nonlinear) excitation field, however, a uniform standing wave is observed between the grating and the CPW, and coupling through the FIB region is strong (Fig. 4b). Left from the CPW the wavelength change of the spin waves can be observed as they decay due to magnetic damping. Beyond about 50 µm propagation self-modulational instability is also causing spikes in the waveform^18^. 2D simulations also confirmed the operation of the device (Fig. 4c). Here the diffraction angles match perfectly the theory, but the position of the focal points are also somewhat behind the Rowland circle. This is the same effect we observe in the experiments. Gratings with larger radius and shorter width would probably not suffer from this deviation, especially at small diffraction angles. However, the position of the peaks is predictable, so this does not affect the usability of the device.

An additional, very unusual aspect of this nonlinear excitation can be observed in Fig. 2c. In case of the direct excitation mechanism one would expect that segments of the grating that are closest to the CPW will excite the highest amplitude waves, since the field of the CPW decays with the lateral distance. However, here we observe exactly the opposite: the strongest “beams” seem to form on the farthest parts of the grating, and amplitudes are less strong in the middle part, which is very close to the CPW (Fig. 3c). This is because the largest area where homogeneous oscillations can occur are on the sides, where there is enough distance between the CPW and the grating. The larger the area, the higher are the dipole fields, and the higher the excitation on the opposite side of the grating. Thus, the discovered indirect excitation mechanism is in comparison very efficient at exciting short-wavelength spin waves on a finely patterned edge at a distance from a straight (or slightly curved) waveguide. This can be advantageous in applications where a complex wavefront has to be launched.

A significant limitation of the nonlinear excitation method is that it only works for single-frequency excitations. If multiple frequency components are excited, the uniform standing waves cannot form, moreover nonlinear mixing creates unwanted spectral pollution. If multiple frequencies are present in the excitation signal (as it is often desirable in a spectrum analyzer), the nonlinear method cannot be used, but, as we demonstrated, the proposed method is very effective at creating devices with complex interference patterns at a single frequency.

## Methods

### Sample fabrication and characterization

YIG thin films were deposited on a GGG substrate using rf-magnetron sputtering (we used 100 nm thick films in the grating experiments). Their magnetic properties were evaluated by means of ferromagnetic resonance (FMR), revealing a saturation magnetization of $\mathrm {M_{s}}$=120 kA/m and a damping constant of $\mathrm {\alpha _{YIG}}=4.4 \times 10^{-4}$. For the excitation of spin waves shorted aluminum CPW antennas were fabricated on top of the YIG film. The antennas were wire bonded to a PCB based CPW with connections to an RF signal generator (Stanford Research SG 386).

The gratings were fabricated in YIG next to the CPW via FIB irradiation. We used 50 keV Ga$^+$ ions with a relatively low dose ($10^{15}$ ions/cm$^2$), which is high enough to almost completely destroy the magnetic properties of YIG, but no material is removed. Much higher doses (in case of ion milling) would likely deteriorate the YIG film around the patterned region due to ion scattering, which makes this method more suitable. Sub-micron resolution patterning can easily be achieved (possibly down to 100 nm in our facility). A similar method was recently described in^19^, where comparably lower doses were used to change magnetic properties of YIG on film level. Here, we deliberately used higher doses to drastically reduce the saturation magnetization of YIG locally, to create a region which inhibits spin-wave transmission. We found that this method is in effect very similar to actually removing material, as it was proposed in^14^.

**Figure 5:**
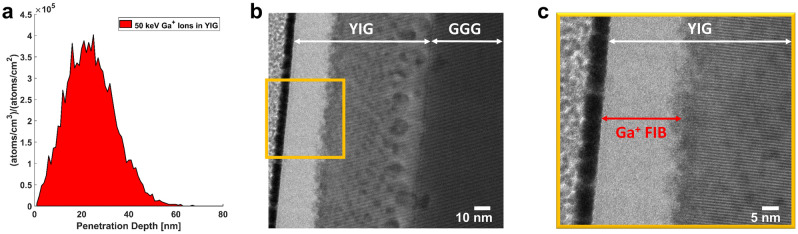
Crystal investigation of the FIB impact in YIG by means of TEM. (**a**) Shows the simulated ion implantation depth for 50 keV Ga$^+$ ions in Y_3_Fe_5_O_12_. A cross-sectional image of a 80 nm thick irradiated YIG film is depicted in (**b**). (**c**) The magnification of the orange square in (**b**) exposes an amorphous toplayer of the thickness expected from the SRIM simulations in (**a**).

The effect of FIB irradiation was also investigated using transmission electron microscopy (TEM). In Fig. 5b,c TEM images indicate that the crystalline structure of YIG is completely destroyed down to a depth of approximately 25 nm, and further significant damage is observable at even higher depths. These results are in good agreement with the previously performed SRIM simulations of our system in Fig. 5a, suggesting a peak implantation depth of 24 nm.

Ga$^+$ ions are relatively large compared to other frequently used ions such as He$^+$, which explains their low penetration depth and the resulting bilayer formation in YIG. We only had access to Ga$^+$ FIB, but SRIM simulations suggest that higher implantation depths could be achieved with He$^+$ ions, and, with higher doses compared to Ga$^+$, similar modification of YIG properties could be achieved with better uniformity across the film thickness.

### Imaging of 2D spin-wave patterns

To image spin-wave interference patterns we built a time-resolved magneto-optical Kerr microscope (trMOKE). Since the Rowland spectrometer requires isotropic spin-wave propagation (as in optics), we had to use out-of-plane bias. In principle slightly anisotropic waves could also be used, as investigated e.g. in^20^, but this requires a redesign of the Rowland grating shape. With the requirement of an out-of-plane bias, we designed our trMOKE to measure the longitudinal Kerr effect, i.e. it is sensitive to changes in the in-plane magnetization component that lies in the incidence plane of the laser^21^. We used a ps-laser with 50 ps pulse width and 405 nm wavelength (PicoQuant Taiko PDL M1 with LDH-IB-405 laser head). With this, we can measure spin waves up to approximately 5 GHz frequency (with 10 MHz steps) and down to 2 µm wavelength. We scan through the sample with an XYZ stage with 0.4 µm resolution. Larger area scans (such as the ones presented in this paper) take about a few hours of measurement time. We use a stroboscopic technique in which the excitation signal is phase locked to the lock-in amplifier and the ps-laser, thus we can extract phase information as well. The amplitude scale is not calibrated, but we estimate that the setup is sensitive to a few percent change in in-plane magnetization.

Currently our setup uses a permanent magnet under the sample for biasing. This makes calibration challenging due to the inhomogeneous field profile. The bias field values in the measurements are approximate values with a few mT uncertainty, and perfect out-of-plane biasing is difficult to achieve.

The spin-wave amplitude and phase were recorded using the following measurement parameters: scanning time step 500 ms, at a sampling rate of 26.16 Samples/s, the step size in x and y direction was 0.8 µm (2 microsteps of the stage). To correct for potential errors in the step size, the recorded data had to be stretched/compressed slightly to fit the applied FIB masks (red overlay) on the grating in the recorded data. The deviation is still small, after fitting a step size of 0.38 µm was used in the X direction instead of the assumed 0.4 µm . This fitting is required due to a possible step-size difference in the X and Y axis of either the trMOKE or the FIB stage. Furthermore, a slight rotation of the recorded pattern was also required due to alignment errors when mounting the sample in the microscope. No image enhancement techniques were applied on the raw data.

### Micromagnetic simulations

Micromagnetic simulations were performed in mumax3^17^. We used experimental values for parameters where they were available ($\mathrm {M_{s}}$=120 kA/m and $\mathrm {\alpha _{YIG}}=4.4 \times 10^{-4}$, 100 nm thickness), and values from literature where we could not directly measure parameters ($\mathrm {A_{ex}}=3.65\times 10^{-12}$ J/m). For discretization we used 30 nm$\times$30 nm$\times$100 nm cells, i.e. approximating the film by a single layer. The lateral cell size is somewhat larger than the exchange length $l_{ex} = \sqrt{2A_{ex}/(\mu _0M_s^2)}\approx {}20$ nm, but it is still at least a hundred times smaller than the wavelength, and no discrepancies could be observed compared to smaller cell sizes, while the simulation can be completed in a reasonable time. To avoid reflections, in the left and right hand side of the simulation 3 µm wide artificial absorbing layers were created using a quadratically increasing damping constant. On the lateral boundaries periodic boundary conditions (single repetition) were used to simulate a long waveguide and avoid energy loss on the sides. The external field was chosen to be 221 mT, using an analytical dispersion formula to match the measurement wavelength at the given frequency (the external field at the exact position of the sample cannot be measured with sufficient precision in our setup). The FIB irradiation was modeled as a region with ($\mathrm {M_{s}}$=0 A/m. The field of the CPW was calculated by HFSS, assuming 1 mA current (peak) in the waveguide. The simulation was run for 180 ns, which was long enough to form a steady interference pattern.

## Conclusions

Optically inspired spin-wave devices represent a promising route towards wave-based computing, which itself is an approach for post-von-Neumann computing. The Rowland grating we demonstrate here is a complex spin-wave optics device and the main component of the Rowland circle spectrometer proposed in^14^. The spin-wave patterns we observe behave remarkably similar to expectations and to the behavior of ideal isotropic waves.

Our device cannot yet operate as a fully functional Rowland spectrometer, due to the nonlinear excitation process that launches the spin waves. The standing waves in the waveguide region form only if a single-frequency excitation is applied at the waveguide. Simulations also confirm that spin waves are not launched if multiple frequency components are simultaneously applied on the waveguide and the device cannot yet perform spectral analysis of a signal with a multitude of frequency components. The device, however, allows the identification of a single applied frequency - this can be determined from the position focus on the Rowland circle. It is likely that a more sensitive read-out method (possibly micro-BLS) could detect spin waves generated directly by the waveguide and would prove that this device is a fully functional spectrometer.

A key result we demonstrate here is the utility of FIB irradiation to draw patterns in YIG with nanoscale precision but without removing material. This minimizes the damage to the adjacent YIG areas. The irradiated patterns can influence spin-wave propagation and may also act as wave sources nearby a waveguide. The manipulation of magnetic properties via FIB in other material systems is well-established, but we are not aware of fabricated spin-wave elements in YIG using a similar approach.

Besides demonstrating complex spin-wave patterns in YIG films, we also described a newfound way of creating spin waves via nonlinear resonance, a method that exploits high-amplitude standing-wave oscillations to indirectly excite short-wavelength spin waves with complex wavefronts. This method itself may have important device applications as it enables strong coupling between a complex-shaped spin-wave launcher (the Rowland grating) and the simple straight waveguide, in cases where only single-frequency excitation is required.
